# Supplementary material for: Loss of ERβ Disrupts Gene Regulation in Primordial and Primary Follicles
Source: Int J Mol Sci. 2024 Mar 11;25(6):3202. doi: 10.3390/ijms25063202 (PMC10970686; doi:10.3390/ijms25063202)
Supplement: Supplementary file 1 [file ijms-25-03202-s001.zip › ijms-2836324-supplementary.pdf]

Supplementary Table 1. Top 20 differentially expressed genes in $Er\beta^{null}$ primordial follicles

Gene symbol	Full name description	TPM value	Fold change	FDR p-value	Brief function
Av320801	PRAME like 3E	11.15	179.56	0.040	No biological data is available yet
H2ac19	H2A clustered histone 19	115.44	94.12	0.006	DNA binding
Or11a4	Olfactory receptor family 11 subfamily A member 4	12.54	33.31	0.010	G protein-coupled receptor activity
Gm14147	Predicted gene 14147	16.71	31.14	0.001	Nucleotide-binding
Gm5795	Predicted gene 5795	16.06	25.31	0.020	No biological data is available yet
Gm8947	Predicted gene 8947	11.46	23.41	0.030	No biological data is available yet
Gm21103	Predicted gene 21103	15.46	20.89	0.020	No biological data is available yet
Gm12184	Cyclin-dependent kinase 2 associated protein 1 retrotransposed	18.43	18.96	0.030	DNA polymerase binding
Pramel28	PRAME like 28	12.79	18.82	0.030	No biological data is available yet
Or8b41	Olfactory receptor family 8 subfamily B member 41	12.71	18.76	0.000	G protein-coupled receptor activity
Gm49388	Predicted gene 49388	14.58	-22.47	0.001	RNA binding
Nutf2	Nuclear transport factor 2	34.53	-21.45	0.003	Structural constituent of nuclear pore
Fam151a	Family with sequence similarity 151 member A	21.84	-20.12	0.010	No biological data is available yet
Vsx2	Visual system homeobox 2	159.32	-16.35	0.000	RNA polymerase II transcription regulatory region sequence-specific DNA binding
Gm49378	Predicted gene 49378	6.53	-10.86	0.050	No biological data is available yet
Pabpn1l	Poly(A)binding protein nuclear 1-like	26.83	-10.63	0.040	Nucleic acid binding
H3c2	H3 clustered histone 2	183.28	-9.99	0.000	DNA binding
Tead3	EA domain family member 3	94.04	-9.96	0.000	RNA polymerase II cis-regulatory region sequence-specific DNA binding
Dnmt1	DNA methyltransferase (cytosine 5) 1	337.80	-8.16	0.000	DNA binding
Gdpd2	Glycerophosphodiester phosphodiesterase domain containing 2	18.27	-7.56	0.001	Phosphoric diester hydrolase activity

Supplementary Table 2. Top 20 differentially expressed genes in *Erβ*^{null} primary follicles

Gene symbol	Full name description	TPM value	Fold change	FDR p-value	Brief function
Gm5128	PRAME like 3C	105.00	116.22	0.000	No biological data is available yet
Rhox4a2	Reproductive homeobox 4A2	84.46	85	0.030	No biological data is available yet
Gm11757	Predicted gene 11757	44.42	82.51	0.040	No biological data is available yet
Or4x18	Olfactory receptor family 4 subfamily X member 18	42.20	43.1	0.001	Signal transduction
H2bc23	H2B clustered histone 23	59.67	23.61	0.009	Chromatin organization
Gm5798	Predicted gene 5798	43.17	19.16	0.020	No biological data is available yet
Gm45799	Predicted gene 45799	11.76	16.62	0.020	Protein transport
Mageb1	MAGE family member B1	62.59	16.12	0.010	Negative regulation of transcription by RNA polymerase II
Gm20605	Predicted gene 20605	14.96	16.07	0.020	Membrane raft assembly
Vmn1r242	Vomeronasal 1 receptor 242	69.51	15.48	0.020	G-protein coupled receptor signaling pathway
Fam177a	Family with sequence similarity 177 member A	35.28	-390.51	0.020	No biological data is available yet
Gm7903	Prame like 3B	37.47	-276.23	0.020	No biological data is available yet
H4c18	H4 clustered histone 18	273.45	-42.93	0.001	DNA binding
Zfp968	Zinc finger protein 968	22.75	-26.11	0.000	RNA polymerase II cis-regulatory region sequence-specific DNA binding
Gm14288	Predicted gene 14288	41.52	-22.47	0.000	Regulation of DNA-templated transcription
Ott	Ovary testis transcribed	29.51	-21.76	0.009	No biological data is available yet
Map1lc3a	Microtubule-associated protein 1 light chain 3 alpha	42.55	-18.02	0.020	Protein binding
Thoc7	THO complex 7	49.32	-17.39	0.020	RNA Binding
Pigyl	Phosphatidylinositol glycan anchor biosynthesis, class Y-like	42.52	-17.09	0.020	Phosphatidylinositol N-acetylglucosaminyltransferase activity
Derpc	DERPC proline and glycine-rich nuclear protein	18.82	-15.7	0.030	Nucleus
